# Nutritional Status of Children Diagnosed With Autism Spectrum Disorder: A Systematic Review and Meta‐Analysis

**DOI:** 10.1111/jhn.70099

**Published:** 2025-07-24

**Authors:** Afnan Alhrbi, Dimitris Vlachopoulos, Ellie‐Mae Healey, Ahmed Taher Massoud, Christopher Morris, Raquel Revuelta Iniesta

**Affiliations:** ^1^ Children's Health and Exercise Research Center University of Exeter Exeter Devon; ^2^ University of Texas University of Texas Health Science Center at Houston Houston Texas USA; ^3^ Peninsula Childhood Disability Research Unit (PenCRU) University of Exeter Medical School, University of Exeter Exeter Devon UK

**Keywords:** autism spectrum disorder, macronutrients, minerals, nutritional status, vitamins

## Abstract

Autism spectrum disorder (ASD) may impact feeding behaviours, which can affect physical development. We aimed to examine published evidence comparing nutritional status, defined as anthropometry, micronutrient status, and intakes and dietary intake, of children with ASD with those of typically developing children (TDC). Eligibility criteria included any studies that compared nutritional status among children with ASD and TDC. PubMed, Web of Science, Scopus and CENTRAL were searched. We used standardized mean difference (SMD) as an effect size for continuous variables and risk ratio (RR) for dichotomous variables with a 95% confidence interval (CI). Thirty‐two studies with 18,480 children (ASD: 2955, TDC: 15,525) were included in our meta‐analysis. Children with ASD were statistically significantly shorter than TDC (SMD: −0.16, 95% CI [−0.28, −0.04], *I* ^2^ = 7%), but no difference in weight [−0.12, 95% CI (−0.17, 0.92)] and BMI [−0.06, 95% CI (−0.32, 0.20)]. They had decreased intake of protein [−0.34, 95% CI (−0.52, −0.15)] and nearly all lipid‐soluble vitamins: vitamin A (SMD: −0.20, 95% CI [−0.38, −0.02], *I* ^2^ = 37%), vitamin D (SMD: −0.30, 95% CI [−0.53, −0.03], *I* ^2^ = 50%), and vitamin K (SMD: −41, 95% CI [−0.71, −0.10], *I* ^2^ = 0%). Also, children with ASD had a statistically significantly decreased intake of some water‐soluble vitamins like folate, riboflavin, thiamine and niacin. Decreased levels of some micronutrients like vitamin D and folate was also found. Children with ASD are statistically significantly shorter than TDC, which can be explained by the lower protein intake and fat‐ and water‐soluble vitamin status and intake. These findings warrant further longitudinal population‐based studies.

## Summary

- Children with ASD often experience feeding difficulties, which may lead to inadequate nutritional intake and deficiencies.
- Previous studies have suggested potential deficits in many micronutrients among this population, but this has never been examined in a meta‐analysis.
- Children with ASD were found to be statistically significantly shorter than typically developing children.
- Children with ASD had a decreased intake of nearly all lipid‐soluble vitamins, protein, and water‐soluble vitamins like folate, riboflavin, thiamine and niacin lower calcium levels. They also had lower levels of vitamin D and folate.
- There was no statistically significant difference regarding weight, BMI, total caloric intake, carbohydrate intake or fat intake.

## Graphical abstract

A series of meta‐analysis from cross‐sectional studies showed that children diagnosed with Autism Spectrum Disorders were statistically significantly shorter than typically developing children, which was explained by the lower protein intake and fat‐ and water‐soluble vitamin status and intake. These findings warrant further longitudinal population‐based studies.

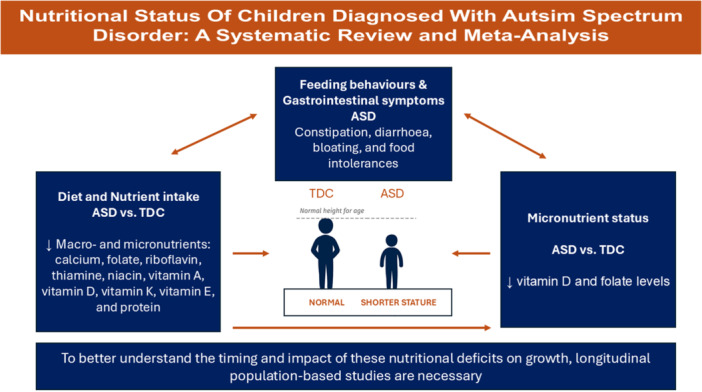

## 1 Introduction

Children with Autism Spectrum Disorder (ASD) usually show limited social and communication skills accompanied by stereotyped repeated behaviours and limited interests [1]. The prevalence of ASD has risen in the last 8 years, particularly in high‐income countries. For instance, in the United States, incidence has increased from 6.7 per 1000 (one in 150) in 2000 to 23 per 1000 (one in 44) in 2018 [2]. ASD is more common in males than it is in females with males being diagnosed approximately three to four times more often than females [3]. Some children with ASD exhibit behaviours such as assaulting, self‐harm, opposition to instructions, and failure to engage in regular speech with a prevalence of 24.10% [2]. As a result, it can be challenging for them to acquire the same quality of life as typically developing children (TDC) [1, 4]. Data suggest that adequate and early intervention can reduce their symptoms and improve their well‐being [5]. Evidence indicates that nutritional and dietary interventions help improve nutritional status, cognitive function, and ASD manifestations [6]. As a result, it is critical to identify the nutritional status, defined as the assessment of anthropometry and growth, micronutrient status, and dietary intake [6], of children with ASD to develop targeted interventions in the future.

Children with ASD tend to have selective eating habits associated with their ASD symptoms with incidence of feeding problems found to be five times higher in children with ASD than TDC [7]. Importantly, increasing evidence report that children with ASD consume less food than the recommended daily intakes, which makes them at higher risk of experiencing faltering growth and essential macro and micronutrient deficiencies in childhood [7, 8, 9, 10]. It is well documented that unhealthy lifestyles including diet increases the risk of non‐communicable diseases in adulthood [11]. Notably, adults with ASD are at higher risk of developing non‐communicable diseases, including type II diabetes, certain cancers, cardiovascular disease and respiratory conditions than the general population. Estimates of the risk of premature mortality in people with ASD are reported to be 16–38.5 years younger on average than the general population [11].

To date one systematic review published in 2013 [7] evaluated evidence from prospective studies comparing anthropometry, feeding problems, and nutrient intake of children diagnosed with ASD compared to TDC; however, plasma or serum micronutrient status was not evaluated. Since then, numerous articles have been published. Thus, we sought to provide an updated systematic review with meta‐analysis, add evidence investigating micronutrient status in children with ASD, and expand the range of study designs. This systematic review and meta‐analysis aimed to evaluate studies investigating the nutritional status of children with ASD in comparison to TDC.

## 2 Methods

### 2.1 Literature Search and Identification of Studies

We designed a protocol a priori, registered under the number (**CRD4202343**). The systematic review is reported according to the Preferred Reporting Items for Systematic Reviews and Meta‐Analyses (PRISMA) guidelines [12]. We comprehensively searched four electronic databases: PubMed, Web of Science, Scopus and CENTRAL, and a nonsystematic search using Google Scholar. This was supplemented by a manual search in the references list of the included studies. References returned by the electronic database searches were imported to the EndNote X9 reference tool and duplicates removed. Two reviewers independently screened the titles and abstracts of the using the inclusion and exclusion criteria. Two reviewers categorized the research as ‘include’, ‘unsure’, or ‘reject’, and their results were compared. Subsequently, two reviewers independently screened full‐text articles for eligibility and agreed the final included list of studies. The search strategy used is shown in Table S6.

### 2.2 Eligibility Criteria

Studies that investigate the dietary intake or dietary habits in children with ASD in comparison to TDC and reporting either anthropometric measures, levels of macronutrients, levels of micronutrients or feeding behaviours were included in this systematic review and meta‐analysis. Randomized control trials (RCT), non‐RCT, observational studies, case‐control studies, case reports, and case series were included.

### 2.3 Data Extraction

Data were extracted independently by two reviewers (AA and AT) using a standardised form created by reviewers based on similar systematic reviews. The following data were extracted from each study: (1) study characteristics: author, year of publication, study design, setting, sample size, and duration of follow‐up; (2) participant characteristics: age, sex, diagnosis of ASD, and comorbidities; (3) nutritional status measures: anthropometric measures, dietary intake, biochemical markers, and feeding practices and (4) outcome measures: changes in nutritional status, associations between nutritional status and ASD symptoms or severity.

### 2.4 Outcome Data

Outcomes summarizing dietary intake and feeding behaviours as well as the anthropometric measurements of children with ASD in comparison to TDC were extracted:

- Anthropometric measurements, including height, weight and BMI.
- Macronutrient intake includes an assessment of total caloric intake, carbohydrate intake, protein intake and total fat intake.
- Water‐soluble vitamin levels, including folate, riboflavin, thiamine, vitamin B12, vitamin B6, vitamin C and niacin.
- Lipid‐soluble vitamin levels, including vitamin A, vitamin D, vitamin E and vitamin K.
- Minerals levels, including calcium, iron, zinc, phosphorus and sodium.

### 2.5 Quality Assessment

Two reviewers (AA and AT) assessed the quality of the included studies and the risk of bias. In cases of disagreement, a 3rd reviewer (RRI) made the final decision. Article quality was examined using the Effective Public Health Practice Project (EPHPP) tool [13]. This involved assessing the included articles according to selection bias, study design, confounders, blinding, data collection methods, withdrawal and drop‐outs and intervention integrity and analyses.

We used the GRADE (Grading of Recommendations Assessment, Development, and Evaluation) guidelines [14] to assess the certainty of evidence of our outcomes [15]. Each outcome was judged to be of high, moderate, low, or very low quality of evidence based on five main domains (1) risk of bias, (2) inconsistency, (3) indirectness, (4) imprecision and (5) publication bias.

### 2.6 Data Analysis

Statistical analysis was conducted utilizing Review Manager Software (RevMan 5.5). The primary metric utilized for evaluating the effects within this study was the standardized mean difference (SMD) along with its 95% confidence interval (CI). The rationale for selecting the SMD as opposed to the mean difference (MD) is attributed to the variation in measurement units across different studies [16]. Some studies measured micronutrient levels using differing units, which could lead to inconsistent comparisons if a simple MD were to be used. The SMD offers a way to normalize these differences by expressing the effect size relative to the variability observed in the studies, thereby enabling coherent and meaningful aggregation and comparison of results despite the heterogeneity of measurement units. *I* ^2^ was used to quantify heterogeneity, with *I* ^2^ > 50% indicating significant heterogeneity. A *p* < 0.05 was considered to indicate statistical significance.

## 3 Results

### 3.1 Study Selection and Characteristics

**Figure 1:**
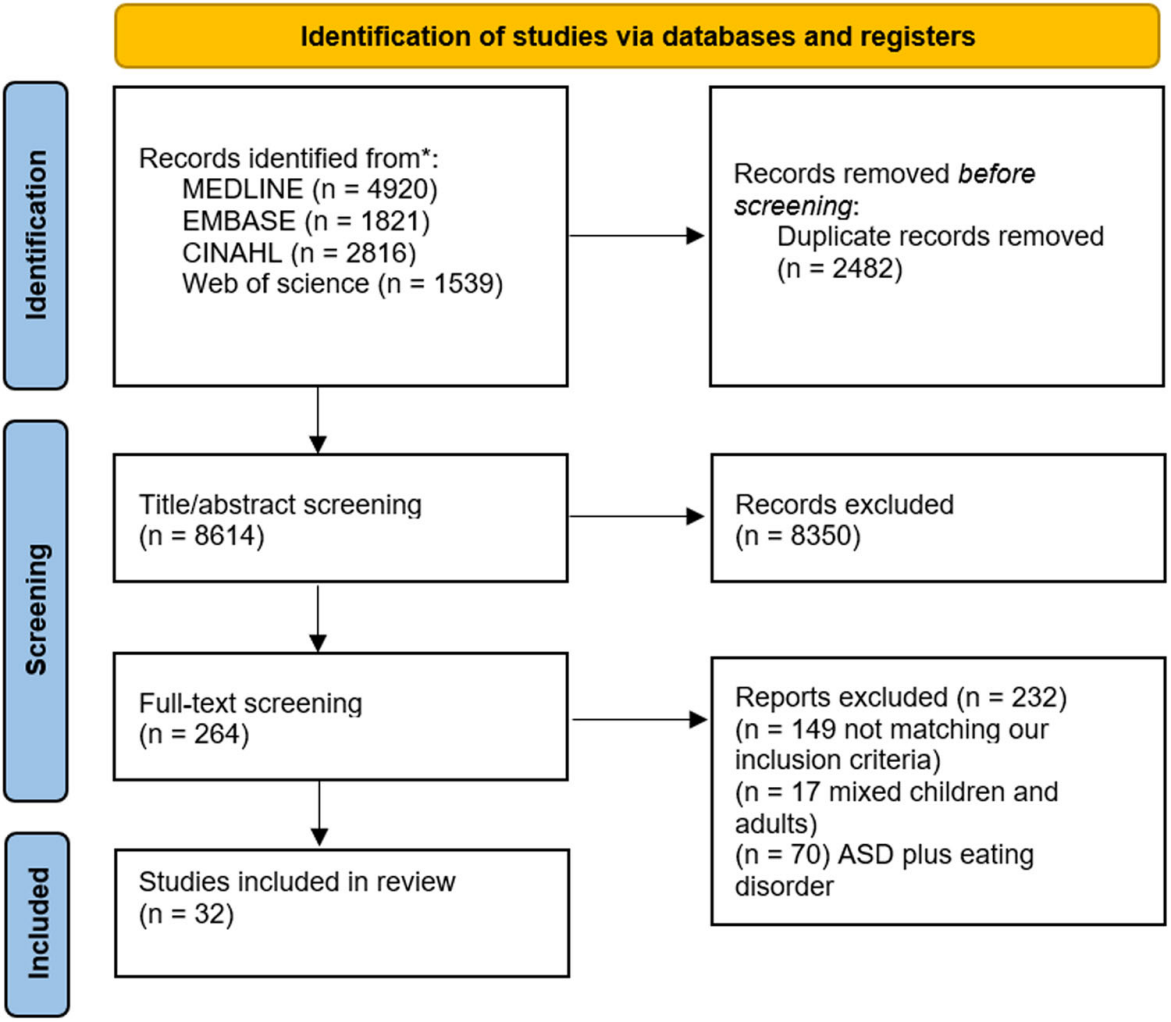
PRISMA flow chart.

The electronic searches retrieved a total of 11,096 records, which were screened according to the eligibility criteria; 32 articles were included (Figure 1).

Four articles were prospective cohort studies [17, 18, 19, 20], nine cross‐sectional studies [21, 22, 23, 24, 25, 26, 27, 28, 29], one clinical trial [30] and 18 case‐control studies [9, 10, 31, 32, 33, 34, 35, 36, 37, 38, 39, 40, 41, 42, 43, 44, 45, 46] (Table 1). The total number of children studied was 18,480 (ASD: 2955, TDC: 15,525). A total of 5239 children were males and 1517 were females. Five studies didn't report the gender distribution [9, 10, 17, 18, 29]. The sample size in the included studies ranged from 24 to 12,980. Regarding the geographical distribution, studies were published in the following locations: China (*n* = 5) [18, 24, 33, 35, 45], from Brazil (*n* = 1) [19], Egypt (*n* = 3) [25, 31, 37], England (*n* = 1) [9], India (*n* = 2) [23, 42], Ireland (*n* = 1) [41], Israel (*n* = 1) [29], orea (*n* = 1) [22], Slovakia (*n* = 1) [20], Spain (*n* = 3) [28, 39, 40], Turkey (*n* = 3) [17, 32, 34] and United States (*n* = 11) [10, 21, 26, 27, 30, 36, 38, 43, 44, 46, 47] (Figure S1).

**Table 1:** Summary of included studies.

ID	Country	Design	Age (years), mean ± SD		Gender (% Male in case/control groups)	Sample size	Two groups	Autism diagnostic criteria nutrition assessment	Main findings
			Cases	Control					
Shearer et al. (1982)	USA	Case‐control	NR	NR	NR	ASD: 12 Control: 12	ASD children, TDC	Definition of the National Society for Autistic Children 3‐Day Food Diary	Children with ASD showed decreased intake of many micronutrients
Schreck and Williams (2006)	USA	Cross‐sectional	8.30 ± 2.50		88%	ASD: 138	ASD children	Gilliam Autism Rating Scale (GARS, Gilliam, 1995) score >= 80 Children's Eating Behavior Inventory (CEBI et al. 1991)	ASD children exhibited peculiar preferences when it came to the foods they were willing to eat. Food preferences reported by their family were connected to the food selectivity of ASD children. More specifically, the child consumed fewer food items in proportion to the family's reported food intake.
Herndon et al. (2008)	USA	Cross‐sectional	4.65 ± 1.16	5.00 ± 1.38	95.7/74.2	ASD: 46 Control: 31	ASD children, TDC	The Autism Diagnostic Observation Schedule‐Generic (ADOS), The Social Communication Questionnaire (SCQ), Autism Diagnostic Interview‐Revised 3‐Day Food Diary	Compared to typically developing children, ASD children ingested significantly more vitamin B6 and E, as well as non‐dairy protein servings, less calcium and less dairy servings.
Lockner et al. (2008)	USA	Cross‐sectional	3.00–5.00 years		Overall: 85%	ASD: 19 Control: 19	ASD children, TDC	NR 3‐Day Food Diary	While some nutrients were ingested in smaller quantities than advised, these were comparable for both typically developing children and children with ASD.
Schmitt et al. (2008)	USA	Case‐control	Range: 7.00–10.00		NR	ASD: 22 Control: 18	ASD children, TDC	NR 3‐Day Food Diary Eating Behaviours Questionnaire	No significant differences in food intake were detected but children with ASD exhibited food behaviours that limited intake of some foods.
Johnson et al. (2008)	USA	Case‐control	3.26 ± 0.75	3.03 ± 0.79	NR	ASD: 19 Control: 15	ASD children, TDC	Autism Diagnostic Observation Schedule (ADOS) Feeding Assessment Survey (FAS) Food Frequency Questionnaire (FFQ) (Yarnell et al. 1983) 24 h Dietary Recall Interview (Johnson et al. 1986)	Results indicated that children with ASD had more mealtime behavioural differences, but these did not translate to significant differences in nutritional status compared to TDC.
Sweetman et al. (2009)	Ireland	Cohort	9.99 ± 4.20	6.43 ± 4.00	88%/56%	ASD: 74 Control: 62	ASD children, TDC	Autism Diagnostic Observation Schedule‐Generic (ADOS‐G)	Zinc and vitamin A mean levels in children with ASD in northwest Ireland were within healthy ranges.
Kim et al. (2010)	Korea	Case‐control	10.10 ± 1.30	10.97 ± 2.70	100%/100%	ASD: 29 Control: 29	ASD children, TDC	DSM‐5	The study indicates the presence of dyslipidemia in boys with ASD and suggest a possibility that dyslipidemia might be a marker of association between lipid metabolism and ASD.
Emond et al. (2010)	England	Cohort	3.75 years		NR	ASD: 79 Control: 12,901	ASD children, TDC	NR	From birth, children with ASD exhibited feeding difficulties and began eating a less diverse diet at the age of 15 months; however, their energy intake and growth remained unaffected. In its most extreme form, feeding behavior in children with ASD can resemble ‘pervasive eating disorder’, reflecting limited interests and trouble accepting change.
Zimmer et al. (2011)	USA	Case‐control	8.20 ± 3.20	8.10 ± 3.30	91%/45%	ASD: 22 Control: 22	ASD children, TDC	ADOS, ADI‐R Harvard Semi‐Quantitative Food Frequency Questionnaire (FFQ)	Children with ASD exhibit significantly lower food variety and are at greater risk for nutritional deficiencies compared to typically developing children.
Evans et al. (2011)	USA	Cohort	6.70 ± 2.40	6.60 ± 2.10	83/78	ASD: 53 Control: 58	ASD children, TDC	Autism Diagnostic Interview‐Revised (ADI‐R) Youth/Adolescent Food Frequency Questionnaire (YAQ) developed from Harvard Food Frequency Questionnaire (Willet, 1998)	Compared to typically developing children, children with ASD consumed significantly fewer servings of vegetables and significantly more juice, sweetened non‐dairy beverages and snack foods. The results showed that while fruit and fruit and vegetable consumption were linked to BMI *z*‐score, juice and sweetened nondairy beverages, snack foods and ‘kids' meals’ were not.
Reynolds et al. (2012)	USA	Cross‐sectional	5.30 ± 2.10		NR	ASD: 222	ASD children, TDC	DSM‐5	Screening for iron stores should be taken into consideration for children with ASD who exhibit symptoms of sleeplessness, inattention, and other medical and behavioural issues that are frequently linked to the disorder.
Sun et al. (2013)	China	Cross‐sectional	4.00–6.00 years		84.9%/84.9%	ASD: 53 Control: 53	ASD children, TDC	DSM‐5 3‐Day Food Diary	Malnutritional deficiencies were noted in both TDC and children with ASD; but were more prevalent in children with ASD.
Bicer and Alsafar (2013)	Turkey	Cross‐sectional	4.00–18.00 years		85%/70%	ASD: 164	ASD children	DSM‐5 Feeding History Questionnaire (The Children's Hospital of Philadelphia 2013) 3‐Day Food Diary	Overweight or obese children made up the majority of the included children with ASD. The majority of children did not consume enough calcium, zinc, vitamin B6 or folate. All age groups consumed a normal intake of salt and cholesterol, while overweight, or obese consumed high cholesterol.
Graf‐Myles et al. (2013)	USA	Cohort	5.72 ± 1.93	4.75 ± 1.83	83%/73%	ASD: 64 Control: 37	ASD children, TDC, non‐ASD developmental delay	Autism Diagnosis Interview‐Revised (ADI‐R; or a Toddler version21), the Autism Diagnostic Observation Schedule (ADOS22) 3‐Day Food Diary	Non‐ASD developmental delay did not differ from the ASD group on consumption of any nutrient, but both were significantly less likely to consume adequate calcium compared to the TDC group. Relative to the TDC group, the ASD group was significantly higher in percent of energy from monounsaturated fats. Children in the ASD group consumed significantly less vitamin A, vitamin D, riboflavin, folate, and calcium than the TDC group. However, only group differences on calcium were associated with inadequate intake The ASD group was significantly less likely to consume at least 100% of recommended dairy servings, and more likely to consume at least 100% of recommended protein servings, than the TDC group, while the non‐ASD developmental delay was less likely than TDC to consume at least 100% of recommended grain servings Children in the ASD group had lower calcium intake and a higher prevalence of inadequate calcium intake compared to TDC.
Hubbard et al. (2014)	USA	Cross‐sectional	6.60 ± 2.1	6.70 ± 2.40	83%/78%	ASD: 53 Control: 58	ASD children, TDC	Autism Diagnostic Interview‐Revised (ADI‐R) Sensory Profile (Ermer et al. 1998) Youth/Adolescent Food Frequency Questionnaire (YAQ) Harvard Food Frequency Questionnaire (Willet, 1998)	No evidence that associations between characteristics of foods and fruit/vegetable consumption differed for children with ASD and TDC (*p*‐values for interactions were all > 0.05) Almost half (49.1%) of children with ASD were reported to always or frequently avoid certain tastes or food smells compared to 5.2% of TD children (*p* < 0.0001) Children with ASD refused foods for more reasons than did TD children
Marí‐Bauset at al. (2015)	Spain	Case‐control	7.01 ± 1.01	8.34 ± 1.19	87%/56%	ASD: 40 Control: 113	ASD children, TDC	Autism Diagnostic Observation Schedule‐Generic (ADOS‐G); the Autism Diagnostic Interview Revised (ADI‐R); and the clinical opinion of an experienced clinical psychologist 3‐Day Food Diary	In ASD, a lower BMI was seen. Vitamin E intakes were higher and fluoride intakes were lower. The lack of diversity in the foods they ate and the insufficiency of some intakes point to the need for regular anthropometric measurements and dietary habits assessment in the monitoring of children with ASD.
Shmaya et al. (2015)	Israel	Case‐control	4.50 ± 0.92	4.30 ± 0.96	80.4%/75.9%	ASD: 50 Control: 12	ASD children, TDC	Autism Screening Questionnaire 3‐Day Food Diary	Children with autism had significantly lower intake of protein, calcium, iron, phosphorus, sodium and zinc.
Saad et al. (2016)	Egypt	Case‐control	5.09 ± 1.42	4.88 ± 1.30	NR	ASD: 122 Control: 100	ASD children, TDC	Childhood Autism Rating Scale (CARS), DSM‐	There is a significant inverse relationship between severity of vitamin D deficiency and ASD rating scales. The mean 25‐OHD levels in patients with severe ASD were significantly lower than those in patients with mild/moderate ASD
Mari‐Bauset et al. (2016)	Spain	Case‐control	7.8 ± 1.23	7.95 ± 1.13	NR	ASD: 105 Control: 495	ASD children, TDC	Diagnostic Observation Schedule‐Generic (ADOSG), the Autism Diagnostic Interview‐Revised (ADI‐R), and the clinical opinion of experienced clinical psychologists. 3‐Day Food Diary	Children with ASD had lower intakes of saturated fatty acids (SFAs) and ω−3 polyunsaturated fatty acids (PUFAs) compared to typically developing (TD) children, but higher total PUFAs, PUFA + MUFA/SFA and ω−6/ω−3 ratios.
Marí‐Bauset et al. (2017)	Spain	Case‐control	7.80 ± 1.23	8.00 ± 1.31	54/60	ASD: 105 Control: 495	ASD children, TDC	Autism Diagnostic Observation Schedule‐Generic (ADOS‐G) and the Autism Diagnostic Interview‐Revised (ADI‐R) 3‐Day Food Diary	Children with ASD failed to meet dietary recommendations for thiamin, riboflavin, vitamin or calcium. Risk of inadequate intake of fibre, vitamin E and sodium was lower in children with ASD than in TDC Children with ASD were at a higher risk of being underweight and of eating more legumes, vegetables, fibre, and some micronutrients (traditional Mediterranean diet) but fewer dairy and cereal products, and less iodine, sodium and calcium than TDC
Liu et al. (2016)	China	Case‐control	5.21 ± 1.83	4.83 ± 0.84	91.6%/91.8%	ASD: 154 Control: 73	ASD children, TDC	DSM‐5 24‐h food weighing method and 2‐Day Food Diary	Reduced macronutrient intakes, severe feeding behavior issues, constipation, and vitamin A deficiency are quite common among children with ASD.
Castro et al. (2016)	Brazil	Case‐control	10.06 ± 3.82	10.02 ± 2.83	100%/100%	ASD: 49 Control: 49	ASD children, TDC	DSM‐5 3‐Day Food Diary	Children with ASD have a higher calorie intake compared to controls, but their diet quality is compromised due to limited food variety and selectivity, leading to nutrient deficiencies. Disruptive mealtime behaviours and a preference for familiar foods further restrict dietary diversity, negatively impacting both the children and their parents.
Zhou et al. (2017)	China	Case‐control	3.80 ± 1.22	3.80 ± 1.22	79%/79%	ASD: 81 Control: 81	ASD children, TDC	DSM‐5	Serum retinoic acid levels were reduced in the group with ASD. Retinoic acid levels were negatively correlated with the severity of ASD.
Malhi et al. (2017)	India	Case‐control	6.11 ± 1.97	6.52 ± 1.93	90.5%/44%	ASD: 63 Control: 50	ASD children, TDC	DSM‐5 The Children's Eating Behavior Inventory (CEBI) Food Frequency Questionnaire (non‐validated)	ASD children had higher children's eating behavior inventory score, more feeding problems, and significantly lower intake of potassium, copper and folate.
Meguid (2017)	Egypt	Case‐control	3.90 ± 0.72	3.70 ± 0.52	78.8/77.5	ASD: 80 Control 80	ASD children, TDC	DSM‐5	Children with ASD had significantly lower dietary and serum levels of calcium, magnesium, iron, folate and vitamin B12 compared to healthy controls. They also had a higher intake of potassium and vitamin C.
Guo (2018)	China	Clinical trial	4.06 ± 1.13	4.24 ± 1.20	51%/86.13%	ASD: 274 Control: 97	ASD children, TDC	Autism Behavior Checklist (ABC), Social Responsiveness Scale (SRS) and Gesell Developmental Scale (GDS)	Children with ASD had vitamin D and folate levels significantly lower than TDC. Children with ASD had significantly lower levels of calcium, magnesium, iron and zinc than TDC, but there were not statistically significant differences in copper levels Correlation analysis showed that vitamin A and calcium levels were negatively correlated with ASD symptoms. Folate, calcium, iron and zinc were positively correlated with the GDS scores in children with ASD
Altun et al. (2018)	Turkey	Case‐control	5.80 ± 2.70	6.70 ± 2.50	86.8%/80%	ASD: 60 Control: 45	ASD children, TDC	DSM‐5	Patients with ASD had significantly lower serum vitamin D and vitamin D receptor (VDR) levels than those in the control group. Nonetheless, children with ASD also had significantly higher homocysteine levels and lower levels of vitamin B6, vitamin B12 and folate.
Buro et al. (2019)	USA	Cross‐sectional	8.00 ± 3.90	8.40 ± 3.80	76%/76%	ASD: 33 Control: 33	ASD children, TDC	NR 24‐h Dietary Recall	Children with ASD had similar HEI‐2015 component scores (55.9) than in NHANES (54.9); however, dairy, whole fruit, vegetables, seafood, and plant protein intake were significantly lower than NHANES data. Fatty acids, refined grains and added sugars were significantly higher in children with ASD compared to NHANES data.
Sengenc et al. (2020)	Turkey	Cross‐sectional	5.95 ± 3.13	6.68 ± 3.80	70%	1529 with ASD ASD: 100 Control: 100	ASD children, TDC	DSM‐5	Children with ASD have significantly lower serum 25‐OHD levels (42.8 ± 19.8) than TDC (48.6 ± 22.4)
Cheng et al. (2020)	China	Case‐control	3 4.72 ± 1.33	4.74 ± 0.90	84.8%/78.9%	ASD: 323 Control: 180	ASD children, TDC	DSM‐5	Children with ASD had lower serum vitamin A levels, and more GI symptoms than TDC. Children with severe ASD had lower vitamin A levels than those with mild and moderate ASD. Compared to ASD children without any GI symptoms, those with total Children with ASD and GI symptoms or constipation had lower vitamin A levels than those without GI symptoms.
Babinska et al. (2020)	Slovakia	Case‐control	6.30 ± 3.60	6.50 ± 3.50	86.2%/53.2%	ASD: 247 Control: 267	ASD children, TDC	Autism Diagnostic Interview‐Revised (ADI‐R), and the Autism Diagnostic Observation Schedule—Second Edition (ADOS‐2) GI symptoms GI severity index questionnaire (Schneider et al. 2006) Usual Diet (method NR)	Children with ASD experience higher prevalence of food selectivity (69.1%) compared to TDC (37.1%, *p* < 0.001). Large proportion of children with ASD displayed food selectivity several times per week or daily compared to TDC (48.8% vs. 13.1%) Anger, requesting food to be prepared in the same way, crying and shouting during meal were significantly more prevalent in ASD than in TDC. 11.4% of children with ASD exhibited aggression during mealtime (TDC no data)

Abbreviations: ABC, Autism Behavior Checklist; ADI‐R, Autism Diagnostic Interview‐Revised; ADOS, Autism Diagnostic Observation Schedule; ASD, Autism Spectrum Disorders; BMI, body mass index; CARS, Childhood Autism Rating Scale; DSM‐5, Diagnostic and Statistical Manual of Mental Disorders, 5th Edition; GI, gastrointestinal; GDS, Gesell Developmental Scale; HEI, Healthy Eating Index; MUFA, Monounsaturated Fatty Acids; NHANES, National Health and Nutrition Examination Survey; NR, not reported; PUFA, Polyunsaturated Fatty Acids; SFAs, Saturated Fatty Acids; SRS, Social Responsiveness Scale; TDC, Typically Developing Children; VDR, Vitamin D Receptor.

### 3.2 Risk of Bias

The majority of the included studies were rated as moderate quality (Table S1). Two studies had a high risk of bias due to lack of blinding, withdrawals and dropout domains [17, 33]. A summary of the quality evidence assessed by the GRADE approach is provided in Table S2.

### 3.3 Meta‐Analysis

Regarding the results of the meta‐analysis, Table 2 shows a description of the statistical analysis of all outcomes. A detailed description of the difference between ASD and TDC as regards nutrient levels and nutrient intake is shown in Tables S3 and S4, respectively.

**Table 2:** Summary of the study outcomes by meta‐analysis.

Outcome	Number of studies	Effect size	*p* value	*I*‐squared
Anthropometric measurements (standardized mean difference)				
Height	6	−0.16, 95% CI [−0.28, −0.04]	< 0.05	7%
Weight	7	−0.12, 95% CI [−0.17, 0.92]	> 0.05	68%
BMI	8	−0.06, 95% CI [−0.32, 0.20]	> 0.05	81%
Macronutrients intake (standardized mean difference)				
Total calories	10	−0.0, 95% CI [−0.35, 0.34]	> 0.05	86%
Protein	14	−0.34, 95% CI [−0.52, −0.15]	< 0.05	57%
Fat	11	−0.20, 95% CI [−0.47, 0.07]	> 0.05	80%
Carbohydrates	11	−0.01, 95% C I [−0.22, 0.20]	> 0.05	54%
Minerals intake (standardized mean difference)				
Calcium	12	−0.78, 95% CI [−1.37, −0.20]	< 0.05	94%
Iron	12	−0.14, 95% CI [−0.62, 0.33]	> 0.05	91%
Zinc	9	−0.16, 95% CI [−0.35, 0.02]	> 0.05	34%
Phosphorus	8	0.20, 95% CI [0.13]	> 0.05	73%
Sodium	6	−0.28, 95% CI [−0.88, 0.32]	> 0.05	90%
Minerals levels (standardized mean difference)				
Calcium	3	0.01, 95% CI [−0.53, 0.56]	> 0.05	86%
Zinc	2	0.01, 95% CI [−0.25, 0.24]	> 0.05	0%
Lipid‐soluble vitamins intake (standardized mean difference)				
Vitamin A	11	−0.20, 95% CI [−0.38, −0.02]	< 0.05	37%
Vitamin D	7	−0.30, 95% CI [−0.56, −0.03]	< 0.05	50%
Vitamin K	3	−0.41, 95% CI [−1.17, 0.12]	< 0.05	0%
Vitamin E	5	0.16, 95% CI [0.03, 0.88]	< 0.05	65%
Lipid‐soluble vitamins levels (standardized mean difference)				
Vitamin D	3	−1.86, 95% CI [−3.63, −0.09]	< 0.05	98%
Water‐soluble vitamins intake (standardized mean difference)				
Folate	9	−0.54, 95% CI [−0.70, −0.39]	< 0.05	93%
Vitamin B6	8	0.29, 95% CI [−0.33, 0.90]	> 0.05	93%
Vitamin C	10	−0.12, 95% CI [−0.51, 0.28]	> 0.05	85%
Riboflavin	9	0.39, 95% CI [−0.67, −0.11]	< 0.05	69%
Vitamin B12	9	−0.65, 95% CI [−1.33, 0.03]	> 0.05	95%
Thiamin	9	−0.19, 95% CI [−0.36, −0.03]	< 0.05	21%
Niacin	10	0.09, 95% CI [−0.08, 0.25]	< 0.05	17%
Water‐soluble vitamins Levels (standardized mean difference)				
Folate	2	1.31, 95% CI [−1.65, −0.98]	< 0.05	99%
Gastrointestinal symptoms (risk ratio)				
Constipation	4	2.79, 95% CI [1.37, 5.66]	< 0.05	85%
Diarrhoea	3	6.70, 95% CI [2.85, 15.76]	< 0.05	0%
Abdominal pain	3	5.09, 95% CI [3.26, 7.96]	< 0.05	0%

#### 3.3.1 Anthropometric Measurements

**Figure 2:**
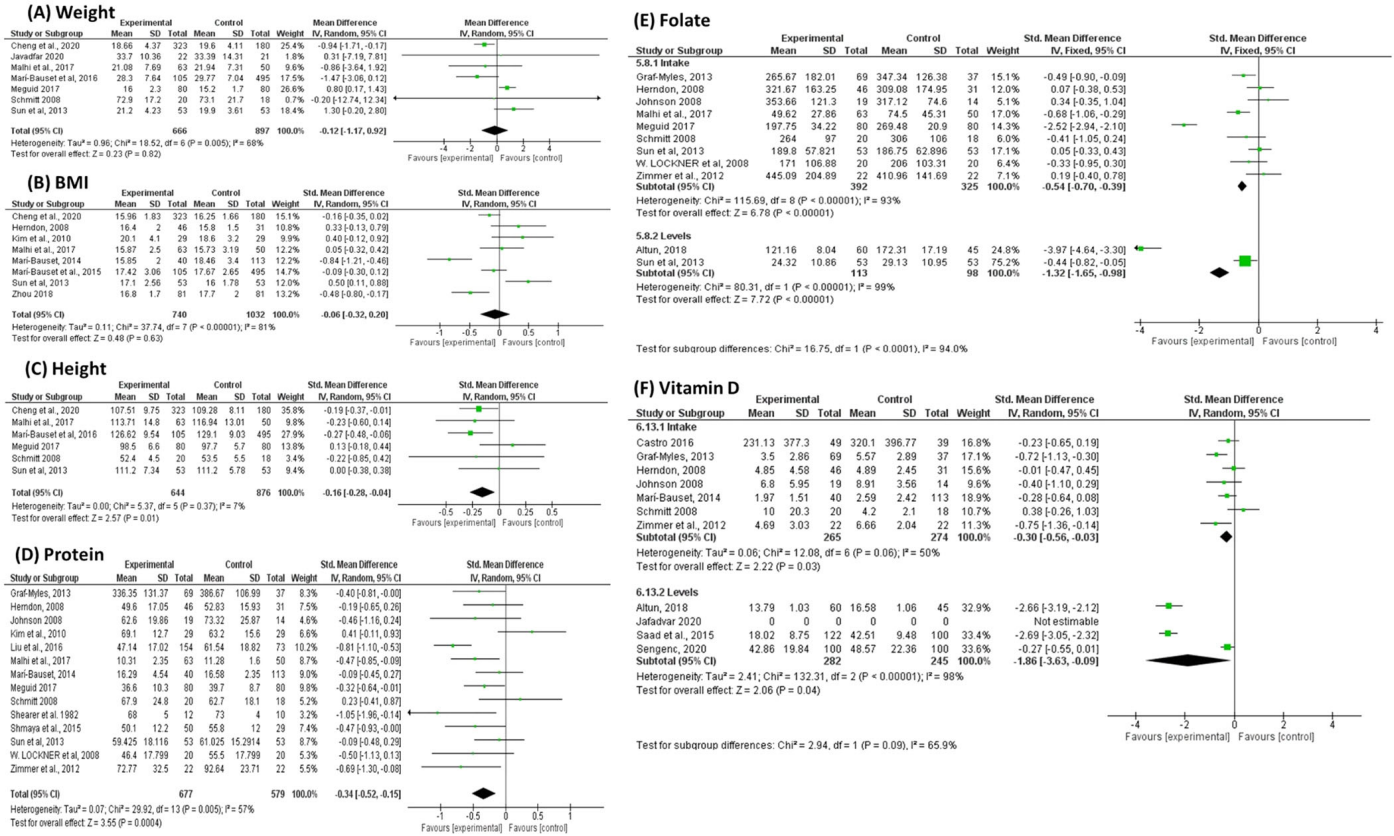
Meta‐analysis from cross‐sectional studies comparing anthrpometry, nutrient intake and vitamin status of children with ASD against TDC. (A) Weight (B) BMI (C) Height (D) Protein (E) Folate (F) Vitamin D.

Children with ASD exhibited a statistically significantly shorter height (SMD: −0.16, 95% CI [−0.28, −0.04], *p* = 0.01; Figure 2C) than did TDC, but there was no significant difference in weight (SMD: −0.12, 95% CI [−1.17, 0.92], *p* = 0.82; Figure 2A) or BMI (SMD: −0.06, 95% CI [−0.32, 0.20], *p* = 63; Figure 2B).

#### 3.3.2 Macronutrients

Although children with ASD had no difference in total energy intake (SMD: 0.00, 95% CI [−0.35, 0.34], *p* = 0.98; Figure S2), they had significantly lower protein intake (SMD: −0.34, 95% CI [−0.52, −0.15], *p *= 0.0004; Figure 2D). There was no difference in carbohydrate (Figure S3), fat intake (Figure S4), or fibre intake (Figure S5).

#### 3.3.3 Water‐Soluble Vitamins

Regarding water‐soluble vitamins, children with ASD had deficiencies in many water‐soluble vitamins, including folate (SMD: −1.32, 95% CI [−1.65, −0.98], *p* < 0.00001; Figure 2E), riboflavin (SMD: −0.39, 95% CI [−0.67, −0.11], *p *= 0.0006; Figure S6), and thiamine (SMD: −0.19, 95% CI [−0.36, −0.03], *p *= 0.02; Figure S7). Nevertheless, there were no significant differences in the following vitamin intakes: vitamin B12 (Figure S8), vitamin B6 (Figure S9), vitamin C (Figure S10), or niacin (Figure S11).

#### 3.3.4 Lipid‐Soluble Vitamins

We found statisitically significant differences in the intake of all lipid‐soluble vitamins. The intake of vitamin A (SMD: −0.20, 95% CI [−0.38, −0.02], *p* = 0.03; Figure S12), vitamin D (SMD: −0.30, 95% CI [−0.56, −0.03], *p* = 0.03; Figure 2F), and vitamin K (SMD: −0.41, 95% CI [−0.71, −0.10], *p* = 0.001; Figure S13) was statistically significantly lower in children with ASD than in TDC. Interestingly, children with ASD consumed significantly more vitamin E (standard mean difference (SMD): 0.46, 95% CI [0.03, 0.88], *p* = 0.03; Figure S14).

#### 3.3.5 Minerals

The only mineral that was consumed in a statistically significantly smaller amount was calcium (SMD: −0.78, 95% CI [−1.37, −0.20], *p* = 0.009; Figure S15). The intake of other minerals, including iron (Figure S16), zinc (Figure S17), phosphorus (Figure S18), and sodium (Figure S19), did not significantly differ between the children with ASD and the TDC.

#### 3.3.6 Gastrointestinal Symptoms

Gastrointestinal symptoms (GI) have been examined in the context of nutrient intake and nutrient deficiencies in ASD. Studies found that children with ASD had an overall increased risk of GI symptoms like constipation, diarrhoea, and abdominal pain (RR: 4.18, 95% CI [2.28, 7.69], *I* ^2^ = 86%). Subgrouping based on each specific symptom showed consistent results (Figure S20).

### 3.4 Correlations between Nutrient Deficiencies and ASD Symptoms

Table S5 summarises the correlations between nutrient deficiencies and ASD severity. Four studies reported that Vitamin A showed significant correlations with symptoms of autism [30, 36, 39, 41]. Specifically, it was found that vitamin A negatively correlated with childhood autism rating Scale (CARS) score with a correlation coefficient ranging from −0.22 to −0.296. One study reported a significant correlation between vitamin D levels and CARS score with a correlation coefficient of −0.50 [31]. Finally, one study reported calcium was significantly correlated with social responsiveness scale (SRS) score with a correlation coefficient of −0.25 [30].

## 4 Discussion

This comprehensive meta‐analysis evaluated the nutritional status (anthropometry, micronutrient status and dietary intakes) of children with ASD compared with TDC. We found that children with ASD consumed statistically significantly lower amounts of protein, calcium, vitamin A, vitamin D, vitamin K, folate, riboflavin, thiamine and niacin. In terms of nutrient levels, children with ASD exhibited significantly lower levels of folate and vitamin D. Most studies, not included in the meta‐analysis, found that deficiencies of some micronutrients like vitamin D and vitamin A were correlated with increased severity of ASD, highlighting that decreased intake does not only impact nutrient levels but also may lead to more pronounced ASD symptoms.

Our meta‐analysis revealed that children with ASD were significantly shorter than TDC (Figure 2C); however, no differences were found in weight or BMI between the groups. To our knowledge, this is the first meta‐analysis to report a statistically significant difference in height between children with ASD and typically developing controls. While some individual studies have noted this trend, it has not previously been quantified in a pooled analysis, making our finding a novel contribution to the literature. We hypothesise that this difference in height is likely the result of long‐term nutritional deficiencies, as indicated by lower intakes of protein and micronutrients (e.g., calcium and vitamin D), as well as poorer micronutrient status. These deficiencies may impair linear growth velocity, with compensatory adaptations in weight and BMI over time and at critical timepoints during childhood growth [48].

Importantly, the comparisons in our meta‐analysis were based on cross‐sectional studies. To better understand the timing and impact of these nutritional deficits on growth, longitudinal population‐based studies are necessary. These studies will help pinpoint when nutrient deficiencies occur during development and guide the design of targeted preventative strategies. In agreement with our results, the meta‐analysis of Sharp et al. found that there was no statistically significant difference between the ASD group and the TDC in BMI [49]. In contrast to our findings, several studies have reported a significantly higher BMI among children with ASD compared to TDC. For example, Castro et al. found that 18.36% (*n* = 9) of their healthy participants were obese, whereas the prevalence of obesity was significantly higher 36.73% (*n* = 18) among children with ASD (*p* = 0.032) [38]. Similarly, Meguid et al. [43] reported a significantly higher BMI/age *z*‐score in the ASD group (0.75 ± 0.9) compared to the TDC group (0.39 ± 0.7; *p* = 0.003). Moreover, Sun et al. found that children with ASD had a higher BMI (17.1 ± 2.56) than TDC (16 ± 1.78) [21]. These discrepancies may be explained by differences in population characteristics, sample size, ASD heterogeneity, cultural and socioeconomic dietary habits, physical activity and unreported medication use such as antipsychotics, which are known to cause weight gain [1]. When aggregated, the pooled evidence suggests that there is no consistent significant difference in BMI or weight between children with ASD and TDC, highlighting the importance of large‐scale meta‐analysis in drawing generalisable conclusions.

This systematic review found that children with ASD exhibited significantly lower intake and levels of many macronutrients and micronutrients, including minerals, water‐soluble vitamins and lipid‐soluble vitamins and these findings have been reported in many previous studies, consistent with our findings [50, 51]. The reasons for decreased intake and, therefore, deficiency in some of these nutrients have been examined in many previous studies [9, 24, 37, 42]. It has been reported that children with ASD often exhibit lower intake of certain nutrients due to sensory sensitivities and selective eating habits. Their heightened sensitivity to food textures, tastes and smells, along with rigid eating preferences, can limit dietary variety and lead to nutrient deficiencies [9, 37, 42]. In addition to abnormal feeding behaviour, multiple studies have found that children with ASD may exhibit abnormal gastrointestinal (GI) symptoms, and these symptoms can lead to decreased intake. In our systematic review, we found that children with ASD had a significantly increased risk of constipation, diarrhoea and abdominal pain. Previous studies have suggested that these symptoms could occur due to abnormal gut microbiota and gut‐brain axis resulting from reduced fibre intake and restricted diet [52]. Surprisingly, the meta‐analysis did not find statistically significant differences in fibre intake between children with ASD and TDC. This finding may reflect the predominance of studies from the United States, where fibre intake tends to be low across the paediatric population (USDA, 2020), potentially masking differences that may be more apparent in populations with higher baseline fibre consumption.

It is plausible that the abnormal feeding behaviour combined with GI symptoms may result in some micronutrients deficiencies due to reduced intake. Many studies suggested that nutrient deficiencies are associated with increased severity of ASD, measured by multiple scales like CARS. For example, Guo et al. found that a deficiency of vitamin A and calcium was associated with increased severity of ASD. Similarly, Altun et al. found that vitamin B6, folate, vitamin B12 and vitamin D deficiencies are all associated with increased severity of ASD. Interestingly, Saad et al. found that vitamin D deficiency led to increased severity of ASD, and administration of vitamin D supplementation to vitamin d‐deficient children is associated with improved CARS scores. The reason for these findings is that certain nutrients, like vitamin A, play a key role in neurodevelopment by regulating neurogenesis and GABAergic signalling, both of which are impaired in ASD. Vitamin A also protects neurons from oxidative stress and inflammation, which are elevated in ASD [53]. Additionally, it influences ASD‐related genes like RORA and enhances oxytocin signalling, which is crucial for social cognition. While vitamin A supplementation may improve gut microbiota and biomarkers in children with ASD, the relationship between retinoic acid (RA) levels and ASD progression remains unclear and requires further research to establish causality [39]. Studies have found similar associations in vitamin D. It was suggested that vitamin D's anti‐inflammatory properties, including inhibiting proinflammatory prostaglandins and NF‐κB, may explain its potential role in reducing ASD severity. Additionally, vitamin D may raise seizure thresholds, increase T‐regulatory cells, protect mitochondria, and enhance glutathione levels, which detoxify oxidative byproducts and chelate heavy metals. These combined effects suggest vitamin D's potential in mitigating the severity of ASD [54]. Folate, like vitamin D, plays an essential role in neurodevelopment by supporting DNA methylation, and neurotransmitter synthesis. Its deficiency has been linked to impaired brain function and altered epigenetic regulation, both of which may contribute to ASD pathophysiology. Additionally, folate is crucial for homocysteine metabolism and antioxidant defence, which are disrupted in children with ASD [55].

Our findings have direct clinical implications, highlighting the need for nutritional screening and targeted interventions in children with ASD. Despite no differences in total energy intake, children with ASD showed significantly lower intake of protein, calcium, folate, and vitamins A, D and K. These deficiencies may be driven by selective eating behaviours and gastrointestinal symptoms common in ASD. While the included studies did not consistently report on supplementation status, our results support the potential value of individualised nutritional assessments and targeted supplementation. Future research should investigate the effectiveness and safety of standardised supplementation protocols for this population, ideally stratified by geographic and dietary context [56].

## 5 Strengths and Limitations

A limitation of this review is that it included observational studies and that there was heterogeneity within the outcomes. This heterogeneity arises from differences in the scales used to assess ASD severity of symptoms and nutritional intake, like 24‐h recalls, food frequency questionnaires and dietary records. These methodological differences may introduce measurement bias and affect the comparability of reported nutrient intake estimates. Also, some studies included children who were on restriction/avoidance diets, which may have had an impact on reported intakes. Moreover, there was also variation in the different age groups and medication use was often non‐reported in the included studies which might be a confounding factor affecting the generalisability of the results. Furthermore, the predominance of cross‐sectional studies in our meta‐analysis limits the ability to establish causal relationships between nutritional deficiencies and ASD outcomes, as temporal directionality cannot be determined. Finally, the majority of the included studies originated from the United States, followed by China. This geographic concentration has important implications for interpreting the results, particularly regarding diet and nutritional status in children with ASD. Dietary patterns, cultural norms, food availability, and access to healthcare differ markedly between and within regions, and these factors likely contributed to the observed variation in nutritional intake and status. Such regional disparities introduce potential confounding and limit the generalisability of pooled estimates [57]. Future studies or meta‐analyses with adequate data may benefit from regional subgroup analyses to more accurately reflect these contextual influences and enhance the applicability of findings across diverse populations.

The strengths of this study include the comprehensive overview of nutrient status in children with ASD, examining both micro‐ and macronutrient intake and how deficiencies contribute to lower levels. The study also explores factors leading to decreased intake, such as gastrointestinal symptoms. These deficiencies collectively result in lower nutrient levels, abnormal anthropometric variables like height, and, most importantly, increased ASD severity. Therefore, this systematic review study highlights the importance of a comprehensive nutritional approach for children with ASD and the need to address these deficiencies to potentially improve symptoms. In addition, to our knowledge, this is the most comprehensive meta‐analysis to date examining the nutritional status of children with ASD in terms of the number of studies included. Compared to previous reviews, our study is the first to meta‐analyse anthropometric outcomes such as height, weight and BMI across studies. Furthermore, it is the first to quantitatively pool correlation coefficients between micronutrient levels and ASD symptom severity, providing a more nuanced understanding of nutritional influences on ASD. Our included studies span from 1982 to 2023, offering a wide temporal lens on the evolving evidence base [58, 59].

## 6 Conclusion

The present systematic review found that children with ASD had significantly lower height compared to TDC, despite no significant differences in weight or BMI *Z* scores. This difference in height may be linked to inadequate intake of essential macro‐ and micronutrients, including calcium, folate, riboflavin, thiamine, niacin, vitamin A, vitamin D, vitamin K, vitamin E and protein. Consequently, children with ASD also exhibited lower levels of vitamin D and folate, which play a crucial role in growth. Many children with ASD experienced gastrointestinal symptoms, including constipation, diarrhoea, bloating and food intolerances. These conditions can impair nutrient absorption and lead to deficiencies in essential vitamins and minerals. Therefore, early detection or even prevention of these deficiencies could help alleviate ASD symptoms and promote optimal growth. However, to better understand the timing and impact of these nutritional deficits on growth, longitudinal population‐based studies are necessary. Such research will help pinpoint when nutrient deficiencies occur during development and guide the design of targeted preventative strategies.

## Author Contributions

All listed authors have contributed to the manuscript substantially and have agreed to the final submitted version. **Afnan Alhrbi:** concept, protocol development, search strategy and search, study selection, data extraction, data analysis and meta‐analysis, report writing and reviewing. **Dimitris Vlachopoulos:** concept, protocol development, report reviewing and editing. **Ellie‐Mae Healey:** search strategy and search, study selection, data extraction, report reviewing and editing. **Ahmed Taher Massoud:** study selection, data extraction, report review and editing. **Christopher Morris:** concept, protocol development, report reviewing and editing. **Raquel Revuelta Iniesta:** concept, protocol development, resolved disagreements (study selection and data extraction), report reviewing and editing.

## Ethics Statement

The authors have nothing to report.

## Conflicts of Interest

The authors declare no conflicts of interest.

## 1 Peer Review

The peer review history for this article is available at https://www.webofscience.com/api/gateway/wos/peer-review/10.1111/jhn.70099.

## Supporting information


**Supplementary Figure S1:** Geographic distribution of included studies.
**Supplementary Figure S2:** Total Energy Intake (Kcal).
**Supplementary Figure S3:** Carbohydrates.
**Supplementary Figure S4:** Fat intake.
**Supplementary Figure S5:** Fiber intake.
**Supplementary Figure S6:** Riboflavin intake.
**Supplementary Figure S7:** Thiamin intake.
**Supplementary Figure S8:** Vitamin B12 intake.
**Supplementary Figure S9:** Vitamin B6 intake.
**Supplementary Figure S10:** Vitamin C intake.
**Supplementary Figure S11:** Niacin intake.
**Supplementary Figure S12:** Vitamin A intake and levels.
**Supplementary Figure S13:** Vitamin K intake.
**Supplementary Figure S14:** Vitamin E intake.
**Supplementary Figure S15:** Calcium levels and intake.
**Supplementary Figure S16:** Iron intake.
**Supplementary Figure S17:** Zinc intake and levels.
**Supplementary Figure S18:** Phosphorus intake.
**Supplementary Figure S19:** Sodium intake.
**Supplementary Figure S20:** Gastrointestinal symptoms.
**Supplementary Table 1:** Summary of quality assessment.
**Supplementary Table 2:** Summary of Grade assessment.
**Supplementary Table 3:** Summary of nutrients intake.
**Supplementary Table 4:** Summary of nutrients levels.
**Supplementary Table 5:** Correlation between ASD severity and nutrients levels.
**Supplementary Table 6:** Correlation between ASD severity and nutrients levels.

## Data Availability

Data sharing is not applicable to this article as no new data were created or analyzed in this study. All the data are available through this manuscript and its supporting material.
