# Supplementary material for: Nutritional Status of Children Diagnosed With Autism Spectrum Disorder: A Systematic Review and Meta‐Analysis
Source: J Hum Nutr Diet. 2025 Jul 24;38(4):e70099. doi: 10.1111/jhn.70099 (PMC12290316; doi:10.1111/jhn.70099)
Supplement: Supplementary file 1 — Supplementary Figure S1: Geographic distribution of included studies. Supplementary Figure S2: Total Energy Intake (Kcal). Supplementary Figure S3: Carbohydrates. Supplementary Figure S4: Fat intake. Supplementary Figure S5: Fiber intake. Supplementary Figure S6: Riboflavin intake. Supplementary Figure S7: Thiamin intake. Supplementary Figure S8: Vitamin B12 intake. Supplementary Figure S9: Vitamin B6 intake. Supplementary Figure S10: Vitamin C intake. Supplementary Figure S11: Niacin intake. Supplementary Figure S12: Vitamin A intake and levels. Supplementary Figure S13: Vitamin K intake. Supplementary Figure S14: Vitamin E intake. Supplementary Figure S15: Calcium levels and intake. Supplementary Figure S16: Iron intake. Supplementary Figure S17: Zinc intake and levels. Supplementary Figure S18: Phosphorus intake. Supplementary Figure S19: Sodium intake. Supplementary Figure S20: Gastrointestinal symptoms. Supplementary Table 1: Summary of quality assessment. Supplementary Table 2: Summary of Grade assessment. Supplementary Table 3: Summary of nutrients intake. Supplementary Table 4: Summary of nutrients levels. Supplementary Table 5: Correlation between ASD severity and nutrients levels. Supplementary Table 6: Correlation between ASD severity and nutrients levels. [file JHN-38-0-s001.docx]

**Supplementary Table 1. Summary of quality assessment.**

| **Study ID** | **A) SELECTION BIAS** | **B) STUDY DESIGN** | **C) CONFOUNDERS** | **D) BLINDING** | **E) DATA COLLECTION METHODS** | **F) WITHDRAWALS AND DROP-OUTS** | **Overall** |
| --- | --- | --- | --- | --- | --- | --- | --- |
|  | **Overall** | **Overall** | **Overall** | **Overall** | **Overall** | **Overall** |  |
| **Shearer et al., 1982** | 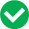 | 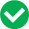 | 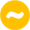 | 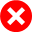 | 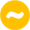 | 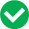 | 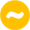 |
| **Schreck and Keith Williams, 2005** | 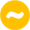 | 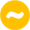 | 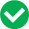 | 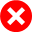 | 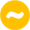 | 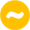 | 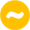 |
| **Herndon, 2008** | 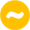 | 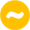 | 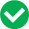 | 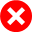 | 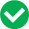 | 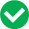 | 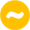 |
| **Johnson, 2008** | 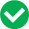 | 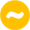 | 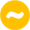 | 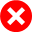 | 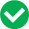 | 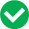 | 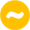 |
| **Schmitt, 2008** | 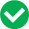 | 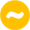 | 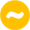 | 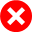 | 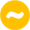 | 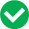 | 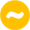 |
| **W. Lockner, 2008** | 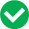 | 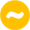 | 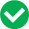 | 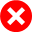 | 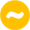 | 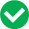 | 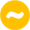 |
| **U. Sweetman,2009** | 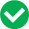 | 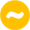 | 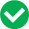 | 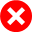 | 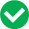 | 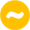 | 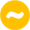 |
| **Emond, 2010** | 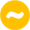 | 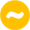 | 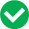 | 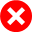 | 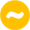 | 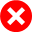 | 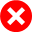 |
| **Kim, 2010** | 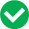 | 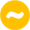 | 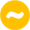 | 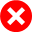 | 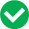 | 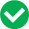 | 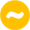 |
| **Evans, 2011** | 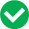 | 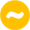 | 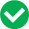 | 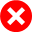 | 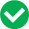 | 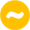 | 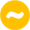 |
| **Zimmer, 2011** | 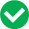 | 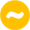 | 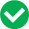 | 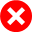 | 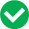 | 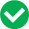 | 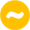 |
| **Reynolds, 2012** | 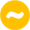 | 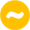 | 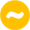 | 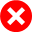 | 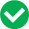 | 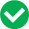 | 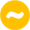 |
| **Bicer and Alsafar, 2013** | 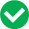 | 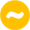 | 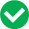 | 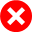 | 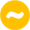 | 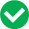 | 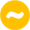 |
| **Graf-Myles, 2013** | 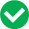 | 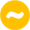 | 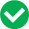 | 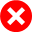 | 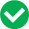 | 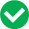 | 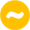 |
| **Sun, 2013** | 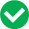 | 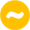 |  |  |  |  |  |
| **Hubbard, 2014** |  |  |  |  |  |  |  |
| **Marí-Bauset, 2014** |  |  |  |  |  |  |  |
| **Shmaya, 2014** |  |  |  |  |  |  |  |
| **Mari-Bauset, 2015** |  |  |  |  |  |  |  |
| **Saad, 2015** |  |  |  |  |  |  |  |
| **Castro, 2016** |  |  |  |  |  |  |  |
| **Liu, 2016** |  |  |  |  |  |  |  |
| **Marí-Bauset, 2016** |  |  |  |  |  |  |  |
| **Malhi, 2017** |  |  |  |  |  |  |  |
| **Meguid, 2017** |  |  |  |  |  |  |  |
| **Zhou, 2017** |  |  |  |  |  |  |  |
| **Altun, 2018** |  |  |  |  |  |  |  |
| **Guo, 2018** |  |  |  |  |  |  |  |
| **W.Buro,2019** |  |  |  |  |  |  |  |
| **Babinska et al., 2020** |  |  |  |  |  |  |  |
| **Cheng, 2020** |  |  |  |  |  |  |  |
| **Sengenc, 2020** |  |  |  |  |  |  |  |

Supplementary Figure 1: Quality assessment of the included studies

strong, Moderate, weak

**Supplementary Table 2. Summary of Grade assessment.**

| **Certainty assessment** | | | | | | | **No of participants** | | **SMD, 95% CI** | **Certainty** |
| --- | --- | --- | --- | --- | --- | --- | --- | --- | --- | --- |
| **No of studies** | **Study design** | **Risk of bias** | **Inconsistency** | **Indirectness** | **Imprecision** | **Other considerations** | **ASD** | **TDC** |  |  |
| **Height** | | | | | | | | | | |
| 6 | 5 Case‒control and 1 cross-sectional | Serious^a^ | Not serious | Not serious | Not serious | None | 644 | 876 | -0.16 (-0.28, -0.04) | ⨁⨁⨁◯ Moderate |
| **Protein intake** | | | | | | | | | | |
| 14 | 10 Case‒control, 2 cross-sectional and  2 cohort | Serious^a^ | Serious^b^ | Not serious | Not serious | None | 677 | 579 | -0.34 (-0.52, -0.15) | ⨁⨁◯◯ Low |
| **Folate intake** | | | | | | | | | | |
| 9 | 5 Case‒control, 3 cross-sectional and  1 cohort | Serious^a^ | Serious^b^ | Not serious | Not serious | None | 392 | 325 | -0.54 (-0.70, -0.39) | ⨁⨁◯◯ Low |
| **Riboflavin** | | | | | | | | | | |
| 9 | 8 Case‒control and  1 cohort | Serious^a^ | Serious^b^ | Not serious | Not serious | None | 402 | 400 | -0.39 (-0.67, -0.11) | ⨁⨁◯◯ Low |
| **Thiamine** | | | | | | | | | | |
| 10 | 8 Case‒control, 1 cross-sectional and  1 cohort | Serious^a^ | Not serious | Not serious | Not serious | None | 386 | 418 | -0.19 (-0.36, -0.03) | ⨁⨁⨁◯ Moderate |
| **Vitamin A intake** | | | | | | | | | | |
|  | 6 Case‒control, 2 cross-sectional and  1 cohort | Serious^a^ | Serious^b^ | Not serious | Not serious | None | 456 | 391 | -0.20 (-0.38, -0.02) | ⨁⨁◯◯ Low |
| **Vitamin D intake** | | | | | | | | | | |
| 7 | 5 Case‒control, 1 cross-sectional and  1 cohort | Serious^a^ | Serious^b^ | Not serious | Not serious | None | 130 | 130 | -0.30 (-0.56, -0.03) | ⨁⨁◯◯ Low |
| **Vitamin K intake** | | | | | | | | | | |
| 3 | 2 case‒control and 1 cohort | Serious^a^ | Not serious | Not serious | Serious^c^ | None | 108 | 69 | -0.41 (-0.71, -0.10) | ⨁⨁◯◯ Low |
| **Vitamin E intake** | | | | | | | | | | |
| 5 | 3 Case‒control and 2 cross-sectional | Serious^a^ | Serious^b^ | Not serious | Serious^c^ | None | 176 | 193 | 0.46 (0.03, 0.88) | ⨁◯◯◯ Very low |
| **Calcium intake** | | | | | | | | | | |
| 12 | 9 Case‒control, 2 cross-sectional and  1 cohort | Serious^a^ | Serious^b^ | Not serious | Serious^c^ | None | 523 | 506 | -0.78 (-1.37, -0.20) | ⨁◯◯◯ Very low |

**SMD;** standardised mean difference, **CI;** confidence interval, **ASD;** autism spectrum disorder, **TDC;** typically developing children.

^a^ Studies were of moderate to weak quality

^b^ Moderate to high heterogeneity was found

^c^ The analysis included a small number of patients, a wide confidence interval, or both.

| **Supplementary Table 3. Summary of nutrients intake.** | | | |
| --- | --- | --- | --- |
| **Study ID** | **Method used to assess intake** | **Measured variables** | **Main findings** |
| Sun, 2013 | A 3 day food diary of their children for two weekdays and one weekend and analyzed by Nutrient calculator software (Fei Hua V2.3, The Institute for Nutrition and Food Security, Chinese Center for Disease Control and Prevention) | Protein, carbohydrates, fat, vitamin A, vitamin B1, vitamin B6, vitamin B2, folate, niacin, vitamin C, vitamin E, calcium, magnesium, iron, zinc | **NR** |
| Herndon, 2008 | 3-Day Food Records and analyzed by Computerized Nutrition Data Systems for Research (NDS-R, University of Minnesota Nutrition Coordinating Center, Minneapolis, MN, USA). | Carbohydrates, energy, fat, fiber, protein, calcium, folate, iron, niacin, tryptophan, tyrosine, vitamin A, vitamin B6, vitamin C, vitamin D, vitamin E, and zinc | **Lower in ASD**: Calcium **Higher in ASD**: Vitamin B6, calcium, vitamin E |
| Marí-Bauset, 2016 | 3-Day Food Records and analyzed by DIAL software to assess the nutritional intake. | Fiber, vitamin B6, folate, vitamin E, vitamin K, vitamin C, vitamin E, thiamin, riboflavin calcium, iron, iodine, zinc, magnesium, sodium, and potassium | **Lower in ASD**: Fiber, vitamin E, and sodium |
| Emond, 2010 | Food Frequency Questionnaire | Iron, zinc, calcium, phosphorus, magnesium, sodium, potassium, iodine, selenium, vitamin A, vitamin C, vitamin D, vitamin E, thiamine, riboflavin, niacin, vitamin B6, folic acid, vitamin B12, energy, carbohydrates, protein, total fat, SFA, MUFA, PUFA, omega-3 fatty acids, cholesterol | **Lower in ASD**: Carotene, vitamin C, vitamin B6, vitamin D |
| Marí-Bauset, 2014 | 3-Day Food Records and analyzed by DIAL software to assess the nutritional intake. | Energy, carbohydrates, total fats, proteins, cholesterol, fiber, folic acid, niacin, vitamin A vitamin B6, vitamin C, vitamin D, vitamin E, biotin, thiamin, riboflavin, vitamin B12, vitamin K, panthothenic acid, calcium, iron, zinc, iodine, phosphorus, fluoride | **Higher in ASD**: Vitamin E |
| Evans, 2011 | Food Frequency Questionnaire | Fruit, vegetables | **Lower in ASD**: fruits and vegetables |
| Lockner, 2008 | 3-day food diary analyzed with Food Processor (version 8.22, 2003, ESHA, Salem, OR) | Iron, calcium, vitamin A, vitamin C, vitamin E, vitamin B6, folic acid, carbohydrates, protein | **Lower in ASD**: Protein, vitamin A, folate, vitamin C, iron |
| Castro, 2016 | 3-day food diary analyzed the software Nutribase® Clinical Edition version 7.18 (USDA, 2006) | Total energy, % calories from protein, % calories from carbohydrates, % calories from total fat, vitamin A, Vitamin B1, Vitamin B2, Vitamin B3, Vitamin B5, Vitamin B6, Vitamin B9, Vitamin B12, Vitamin C, Vitamin D, Calcium, Sodium, Iron, Selenium, Iron, Zinc, Magneisum, Phosphorus, Potassium | **Lower in ASD**: Iron, omega-3 fatty acids. **Higher in ASD**: Total energy, carbohydrates, vitamin B9, calcium, sodium. |
| Johnson, 2008 | 24 h diet analyzed with the software (Food Processor, Version 8.5 2005) | Iron, zinc, calcium, phosphorus, magnesium, sodium, selenium, vitamin C, vitamin D, vitamin K, thiamine, riboflavin, niacin, pantothenic acid, vitamin B6, folic acid, vitamin B12, energy, carbohydrates, protein, total fat, PUFA, omega-3 fatty acids, fibre | **Lower in ASD**: Vitamin K |
| Kim, 2010 | 24 h diet analyzed with Computer-Aided Nutritional Analysis Program (CAN-Pro, version 2.0) developed by the Korean Society of Nutrition (2000) | Total cholesterol, Triglycerides, HDL-C, LDL-C | **Lower in ASD**: HDL-C **Higher in ASD**: LDL-C |
| Liu, 2016 | A combination of a 24-hour food weighing method and two-day diet diaries, conducted over one week. Software not specified | Vitamin A, energy carbohydrates, protein, total fat | **Lower in ASD**: Total energy, protein, fat, and carbohydrates |
| Malhi, 2017 | 3-day food diary. Software not specified. | Iron, zinc, calcium, phosphorus, sodium, potassium, cooper, vitamin A, vitamin C thiamine, riboflavin, niacin, folate, vitamin B12. | **Lower in ASD**: Copper, folate, potassium |
| Meguid, 2017 | 3-day food diary. Software not specified. | Iron, zinc, calcium, phosphorus, magnesium, sodium, potassium, selenium, vitamin A, vitamin C thiamine, riboflavin, vitamin B6, folic acid, vitamin B12, energy, carbohydrates, protein, total fat, fiber. | **Lower in ASD**: Folic acid, vitamin B12, calcium, iron, magnesium, sodium, selenium. **Higher in ASD**: Fibers, vitamin B6, vitamin C, potassium. |
| Mari-Bauset, 2015 | 3-Day Food Records and analyzed by DIAL software to assess the nutritional intake. | Total fat, SFA, MUFA, PUFA, omega-3 fatty acids, cholesterol | **Lower in ASD**: SFA **High in ASD**: PUFAs |
| Schmitt, 2008 | 3-day food diaries analyzed using Food Processor Nutrition Analysis Software (ESHA, Salem, Oragon) | Total energy, protein, carbohydrates, fiber, total fat, vitamin A, thiamin, riboflavin, niacin, vitamin B6, vitamin B12, vimtain C, vitamin D, vitamin E, vitamin K, Biotin, calcium, iron, phosphorus, potassium, selenium, sodium, zinc, iodine, copper, magnesium | No differences |
| Shmaya, 2014 | 3-day food diary. Software not specified. | Protein, carbohydrates, fiber, calcium, Iron, Magnesium, phosphorus, potassium, sodium, zinc, vitamin E, vitamin C, thiamin, riboflavin, niacin, pyridoxine, vitamin B12 | **Lower in ASD**: Protein, calcium, iron, phosphorus, zinc, vitamin B12 |
| ASD: Autism Spectrum Disorder, NR: Not Reported, NDS-R: Nutrition Data Systems for Research, SFA: Saturated Fatty Acids, MUFA: Monounsaturated Fatty Acids, PUFA: Polyunsaturated Fatty Acids, HDL-C: High-Density Lipoprotein Cholesterol, LDL-C: Low-Density Lipoprotein Cholesterol. | | | |

| **Supplementary Table 4. Summary of nutrients levels.** | | |  |
| --- | --- | --- | --- |
| **ID** | **Measured variables** | **Significant variables** |  |
|  |  |  |  |
| Sun, 2013 | Zinc, calcium, iron, vitamin A, folate, vitamin B12 | **Lower in ASD**: Calcium, vitamin A, folate |  |
| Sengenc, 2020 | Calcium, phosphorus, alkaline phosphatase, vitamin D | **Lower in ASD**: Vitamin D **Higher in ASD**: Phosphorus, alkaline phosphatase |  |
| Guo, 2018 | Vitamin A, Vitamin D, Vitamin B12, folate, ferritin, calcium, magnesium, iron, zinc, copper | **Lower in ASD**: Vitamin D, folate, calcium, magnesium, iron, zinc |  |
| Saad, 2015 | Vitamin D | **Lower in ASD:** Vitamin D |  |
| U. Sweetman,2009 | Vitamin A, zinc | **Higher in ASD**: Vitamin A |  |
| Reynolds, 2012 | Iron, ferritin | No control. Many ASD patients had below normal iron and ferritin levels |  |
| Cheng, 2020 | Vitamin A | **Lower in ASD**: Vitamin A |  |
| Altun, 2018 | Vitamin D, vitamin B6, folate, vitamin B12, calcium, phosphorus, alkaline phosphatase | **Lower in ASD**: Vitamin D, vitamin B6, folate, vitamin B12. **Higher in ASD**: Homocysteine |  |
| Zhou, 2017 | Vitamin A | **Lower in ASD**: Vitamin A |  |
| Liu, 2016 | Ferritin, folate, vitamin B12, vitamin D, vitamin A | No control. 2%-77.9% of ASD patients had deficiency of at least one micronutrient |  |

| **Supplementary Table 5. Correlation between ASD severity and nutrients levels.** | | | | |  |
| --- | --- | --- | --- | --- | --- |
| **Study ID** | **Scale used** | | **Nutrients correlated with severity** | **Notes** |  |
| **Saad, 2015** | CARS | | Vitamin D (r = -0.50, p < 0.001) | Administration of vitamin D improved CARS score |  |
| **Liu, 2016** | CARS | | Vitamin A (r = -0.22, p = 0.021) Ferritin (r = -0.017, p = 0.91) Folate (r = -0.25, p = 0.095) Vitamin B12 (r = -0.24, p = 0.12) Vitamin D (r = -0.02, p = 0.88) | **NA** |  |
| **Zhou, 2017** | CARS | | Vitamin A (r = -0.53, p < 0.001) Homocysteine (r = 0.29, p = 0.008) C-reactive protein (r = 0.27, p = 0.012) IL-6 (r = -0.36, p = 0.001) | **NA** |  |
| **Guo, 2018** | SRS | | Vitamin A (r = -0.296, 0.013) Vitamin D (r = -0.068, 0.57) Vitamin B12 (r = -0.131, p = 0.29) Folate (r = 0.11, p = 0.35) Ferritin (r = 0.16, p = 0.18) Calcium (r = -2.5, p = 0.04) Magnesium (r = -0.014, p = 0.94) Iron (r = -0.026, p = 0.90) Zinc (r = 0.09, p = 0.64) Copper (r = -0.049, p = 0.80) | **NA** |  |
| **Altun, 2018** | CARS | | Vitamin D: (r -0.687) Vitamin B6 (r = -0.83) Folate (r = -0.84) Vitamin B12 (r = -0.79) | **NA** |  |
| **Cheng, 2020** | SRS. ABC. CARS | | Vitamin A deficiency patients had significantly higher CRS, ABC, and CARS scores. | **NA** |  |
| ASD - Autism Spectrum Disorder, SRS - Social Responsiveness Scale, CARS - Childhood Autism Rating Scale, ABC - Aberrant Behavior Checklist, CRS - Children's Rating Scale, IL-6 - Interleukin 6, NA - Not Applicable. | | | | |  |
| **Supplementary Table 6. Correlation between ASD severity and nutrients levels.** | | | | | |
| **Search strategy** | | (("macronutrient*" OR "micronutrient*" OR "nutrition intake*" OR "diet intake*" OR "food intake*" OR  "nutrient status" OR "nutritional status" OR "nutritional state" OR "nutritional health" OR  "nutrient balance" OR "nutritional adequacy" OR "nutrition inadequacy" OR "nutrition insufficiency" OR  "nutrient deprivation" OR "malnutrition" OR "undernutrition" OR "overnutrition" OR  "macronutrient* deficiency" OR "micronutrient* deficiency" OR "micronutrient* inadequacy" OR "anthropometric measurement*" OR "body mass index" OR BMI OR  "waist circumference*" OR "hip circumference*" OR "waist/hip circumference ratio*" OR  "waist/height ratio*" OR WHtR OR "BMI-Z score" OR "height-Z score" OR  (MH "Dietary Intake") OR (MH "Feed Intake") OR (MH "Food Intake") OR (MH "Ingestion") OR  (MH "Macronutrient Intake") OR (MH "Micronutrient Intake") OR (MH "Nutrient Intake") OR  (MH "Nutritional Intake") OR (MH "Anthropometry") OR (MH "Body composition") OR (MH "Body Weights and Measures")  AND   ("medical nutrition therapy" OR MNT OR "nutritional counselling" OR "dietary counselling" OR  "diet intervention*" OR "diet therapy" OR "diet education" OR "dietary advice" OR  "dietary strategy*" OR "clinical nutrition" OR "nutritional support" OR  "nutritional intervention*" OR "functional nutrition" OR "integrative nutrition" OR  "holistic nutritional therapy" OR "dietary supplement*")   AND   (toddler* OR preschool OR "preschool child*" OR "school child*" OR "school-age child*" OR  "primary school*" OR "elementary school*" OR "high school*" OR  child* OR kid* OR minor* OR youth* OR teenager* OR adolescent* OR  pubescent OR juvenile* OR youngster* OR pre-adult OR paediatric* OR pediatric* OR  (MH "Minors") OR (MH "Child, Preschool") OR (MH "Adolescent") OR (MH "Pediatrics") OR (MH "Puberty"))   AND   (autis* OR "autism spectrum disorder*" OR ASD OR ASDS OR  "autism spectrum" OR "autism syndrome" OR "autistic spectrum condition*" OR  "autism spectrum behavior*" OR "autism spectrum condition*" OR ASC OR  "pervasive developmental disorder*" OR PDD OR PDD-NOS OR  "asperger syndrome" OR "asperger's syndrome" OR "autistic disorder" OR AD OR  "atypical autism" OR "childhood autism" OR "high-functioning autism" OR  "low-functioning autism" OR "regressive autism" OR "non-verbal autism" OR  "classic autism" OR "Kanner's syndrome" OR "social pragmatic communication disorder" OR SCD OR  "pathological demand avoidance") | | | |

**Supplementary Figure S1. Geographic distribution of included studies.**

**Supplementary Figure S2. Total Energy Intake (Kcal).**

**Supplementary Figure S3. Carbohydrates.**

**Supplementary Figure S4. Fat intake**

**Supplementary Figure S5. Fiber intake**

**Supplementary Figure S6. Riboflavin intake**

**Supplementary Figure S7. Thiamin intake**

**Supplementary Figure S8. Vitamin B12 intake**

**Supplementary Figure S9. Vitamin B6 intake**

**Supplementary Figure S10. Vitamin C intake**

**Supplementary Figure S11. Niacin intake**

**Supplementary Figure S12. Vitamin A intake and levels**

**Supplementary Figure S13. Vitamin K intake**

**Supplementary Figure S14. Vitamin E intake.**

**Supplementary Figure S15. Calcium levels and intake**

**Supplementary Figure S16. Iron intake**

**Supplementary Figure S17. Zinc intake and levels**

**Supplementary Figure S18. Phosphorus intake**

**Supplementary Figure S19. Sodium intake**

**Supplementary Figure S20. Gastrointestinal symptoms.**
